# A dataset of the distribution of aflatoxin-producing fungi in Japan

**DOI:** 10.1016/j.dib.2025.111868

**Published:** 2025-07-11

**Authors:** Yuko Tsukada, Masayo Kushiro, Hitomi Wakatsuki, Toshihiro Hasegawa, Motoki Nishimori

**Affiliations:** a Institute of Food Research, NARO, 2-1-12 Kannondai, Tsukuba, Ibaraki 305-8642, Japan; b Institute for Agro-Environmental Sciences, NARO, 3-1-3 Kannondai, Tsukuba, Ibaraki 305-8604, Japan

**Keywords:** *Aspergillus flavus*, *Aspergillus parasiticus*, Climate change, Global warming, Mycotoxin, Range shift, Systematic review

## Abstract

Mycotoxins are secondary metabolites produced by fungi that can cause adverse effects on animals and humans. Above all, aflatoxin (AF), produced by some species of genus *Aspergillus*, is a potent genotoxic and carcinogenic toxin. Since environmental factors are known to affect the risk of fungal growth and mycotoxin production, it is expected that climate change will increase the contamination of agricultural commodities with mycotoxins. AF-producing fungi have been thought to be distributed mostly in tropical and subtropical regions; however, there is a concern that the distribution of these fungi is expanding in Japan due to climate change. There is a lack of research on the risk of mycotoxin contamination under a climate change in Japan, and studies are needed to predict fungal occurrence and mycotoxin contamination in Japan. Here, we present a dataset collected through a systematic literature search from publicly available data sources (Web of Science, CiNii Research, J-STAGE, AgriKnowledge and NARO Genebank), for distribution of AF producing fungi. This dataset includes information of AF-producing fungi distribution surveyed in 33 of the 47 prefectures and corresponding geographic data. This dataset is useful resources for conducting meta-analyses to quantify the climate change impact on distribution of AF producing fungi in Japan and contribute the risk assessment of AF contamination.

Specifications Table

**Table:** 

Subject	Biology
Specific subject area	Risk assessment of aflatoxin (AF) contamination; climate change impacts on distribution of AF-producing fungi.
Type of data	Table, Figures, Reference list. Raw, Filtered.
Data collection	The presence and absence of aflatoxin-producing fungi in soil and crop samples across Japan were assessed through a systematic review of five databases: Web of Science, CiNii Research, J-STAGE, AgriKnowledge, and the NARO Genebank. Searches conducted from June 2023 to January 2024 used English and Japanese terms related to *Aspergillus*, aflatoxin, distribution, and contamination. Of 450 articles identified, 25 met the criteria, with 14 more added via reference tracking. After removing overlaps, 21 studies remained, yielding 137 distribution records. Site locations were extracted from texts or maps, and 10-year climate averages were obtained from the nearest AMeDAS stations.
Data source location	The distribution of AF-producing fungi was investigated in 33 prefectures, while 14 prefectures have not yet been investigated. All articles provided the data is listed in the reference list file in the repository.
Data accessibility	Repository name: Mendeley Data Data identification number: 10.17632/z5bzhktswx.2 Direct URL to data: https://data.mendeley.com/datasets/z5bzhktswx/2
Related research article	None

## 1 Value of the Data

**Fig. 1:**
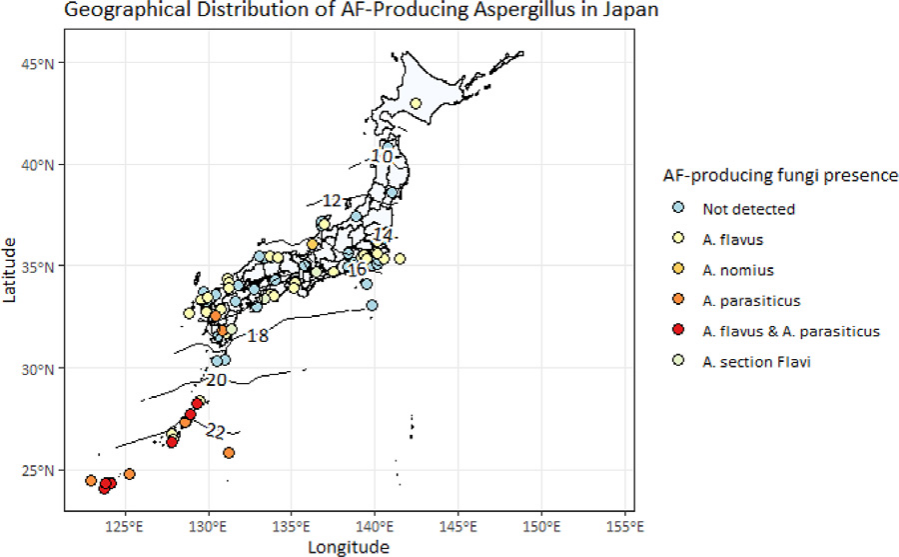
A map illustrating the reported presence and absence of aflatoxin-producing fungi in the dataset. Taxonomic identifications are represented in different colors, and locations where presence was not confirmed are marked as “Not Detected.” Contour lines and accompanying numbers indicate the annual average temperatures (in °C) for the 10-year period surrounding the sampling times, which are also recorded in the dataset. Temperature point data were initially spatially interpolated using the “akima” package in R, and the map was generated using the ggplot function in R. The code to create the map is available in the same repository.
• The data is freely available and reusable without permission under the CC-By 4.0 license, as indicated in the Mendeley repository.

• The dataset includes 137 data for the distribution of AF-producing fungi observed in soil or crop samples in Japan, systematically collected from scientific literature and the NARO Gene bank.
• This data collection also includes information on the year sampling was conducted and corresponding geographic data. Where detailed geographical information is available, we compiled climatological data such as annual temperature and precipitation around the time of sampling. This will allow for an analysis of potential relationships between the distribution AF producing fungi and climatological conditions.
• This dataset offers useful resources for conducting meta-analyses to quantify the climate change impact on distribution of AF producing fungi in Japan.
• The dataset is ready to use in the machine learning application. It can be easily loaded using R code to produce maps showing the distribution aflatoxin-producing fungi and associated climatological data. An example output is shown in Fig. 1, and the R code to generate the map is available at the Mendeley repository, demonstrating how the dataset can be used for spatial analysis.

## 2 Background

Contamination with aflatoxin (AF), a potent mycotoxin, is a significant pathway by which climate change reduces food safety [1]. AF is a type of mycotoxin mainly produced by Aspergillus species, such as *Aspergillus flavus* and *A. parasiticus*, is a potent genotoxic and carcinogenic toxin. These fungi thrive in warm and humid environments, which explains their prevalence in tropical and subtropical regions.

In temperate areas like Japan, AF contamination has mostly been reported in imported foods rather than domestic agricultural products [2]. However, climate change may expand the geographical distribution of aflatoxigenic fungi and amplify AF-related risks [[3], [4], [5]]. While a comprehensive nationwide soil survey of AF-producing fungi was conducted around 50 years ago [6], subsequent surveys have been fragmented and insufficient to capture current trends.

To prevent the risks of AF contamination in food and feed, it is essential to develop a comprehensive scientific dataset that covers the distribution of AF-producing fungi across Japan, both past and present. To this end, we conducted a systematic literature review to collect evidence on the historical and current presence and absence of AF-producing fungi previously examined in Japan. This dataset will enable effective monitoring of fungal distribution as temperatures rise.

## 3 Data Description

The dataset in the Mendeley repository contains the following five files:

(1) “AF_distribution_Dataset_2025.06.22.xlsx” is the main data spreadsheet file containing the values of the variables listed in “Metadata_2025.06.22.xlsx”.
(2) “Metadata_2025.06.22.xlsx” contains metadata for the main dataset, summarizing variable names, units, and data types used in “AF_distribution_Dataset_2025.06.22.xlsx”.
(3) “Reference list.xlsx” provides a list of 39 studies identified from the search on the five scientific databases considered. References selected from the reviewed literature’s bibliography were marked with “⁎” followed by the reference number, with the source reference number indicated in the righthand column of this file.
(4) “Prefecture_dataset.xlsx” provides information on the number of samples and reported studies in each prefecture. Through the search and screening steps, we collected data from 33 out of 47 prefectures, the geographic distribution of which is shown in Fig. 4. While this dataset covers studies assessing the presence and absence of aflatoxin-producing *Aspergillus* species in Japan, approximately 30 % of the prefectures have not been tested or reported, highlighting the data gap.
(5) “AF_Distribution_Japan_map.zip” contains an R script visualizing a distribution map of Aflatoxin-producing fungi along with shapefiles for Japanese administrative boundaries.

**Fig. 2:**
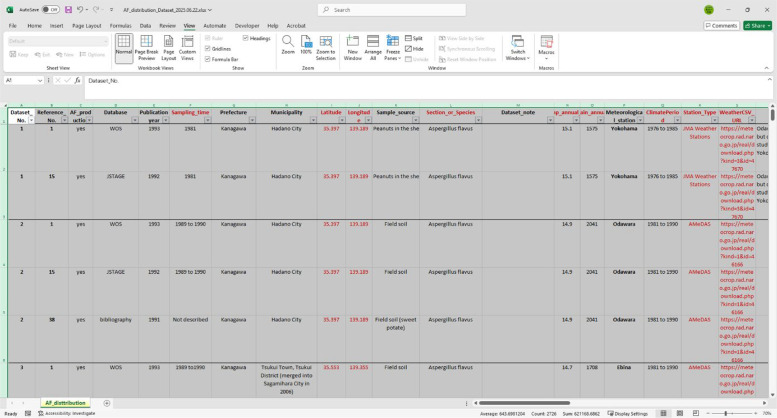
A preview of the main dataset stored in the Mendeley repository, containing results from studies on the distribution of aflatoxin-producing fungi in Japan, collected through a systematic literature and database search.

A preview of the main dataset is shown in Fig. 2.

**Fig. 3:**
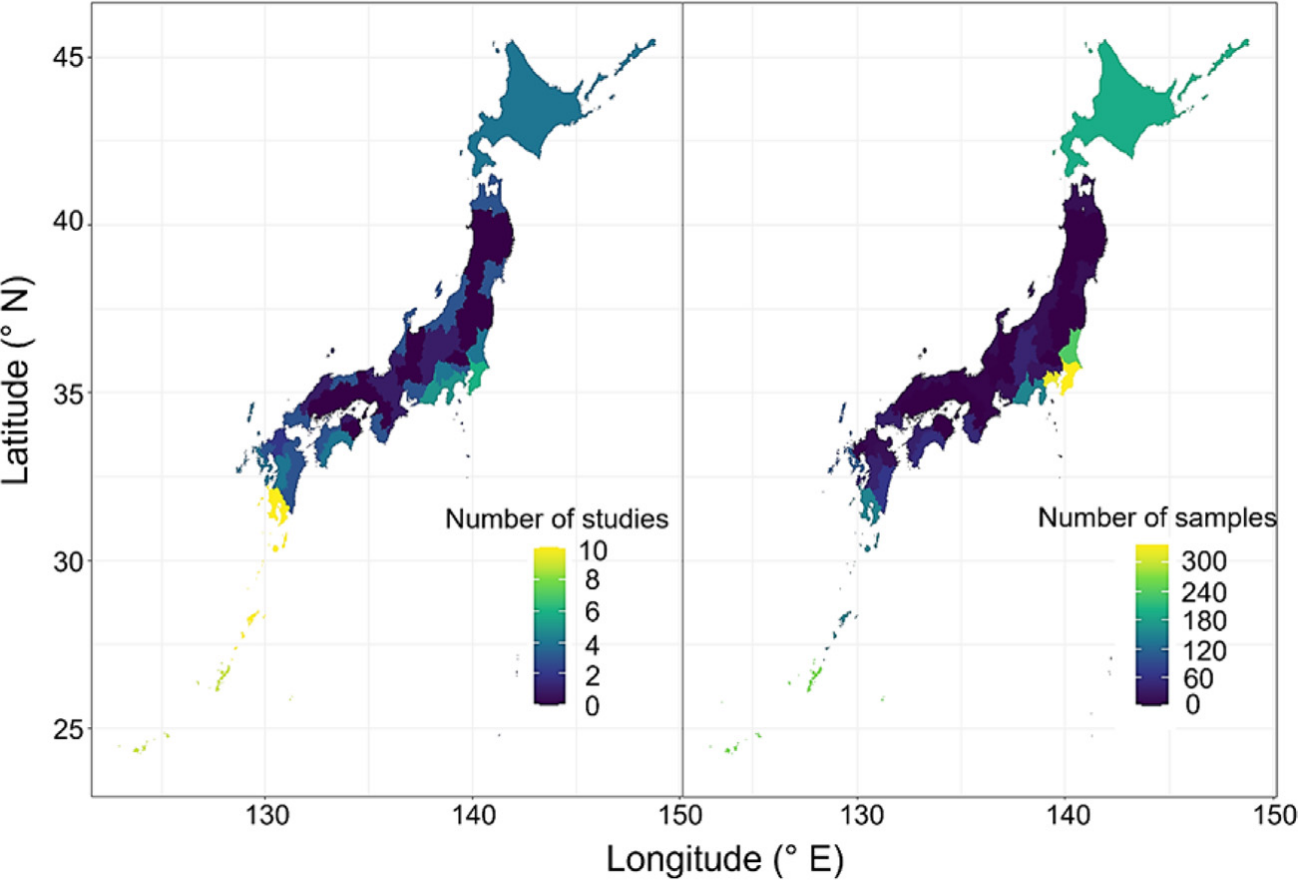
Number of studies reporting the presence or absence of AF-producing fungi in collected samples (left) and the number of samples examined (right) in each prefecture, obtained through a systematic review.

The PRISMA diagram depicting the systematic literature search process is presented in Fig. 3.

## 4 Experimental Design, Materials and Methods

### 4.1 Inclusion and exclusion criteria

We selected studies satisfying the following criteria:

#### 4.1.1 Studies collected from databases

(1) We selected studies with the publication type “journal article”. Reviews and conference abstracts were excluded.
(2) Articles using samples obtained outside Japan were excluded, and articles using samples from Japan were selected.
(3) Articles with soil or crop samples were selected. Articles containing the words “rat, milk, cow, cheese, butter, liver, bird, chicken, or animal” in the title were excluded.
(4) We excluded studies on *Aspergillus oryzae* that does not produce AF [7].
(5) Studies on health hazards, such as aspergillosis, with the words Symptoms, Diseases, or Health were excluded.

#### 4.1.2 References selected from the reviewed literature’s bibliography

(1) Relevant articles in the bibliographies of studies selected through the process described in Section 4.1.1 were selected.
(2) Articles without full text were excluded.

### 4.2 Data collection

**Fig. 4:**
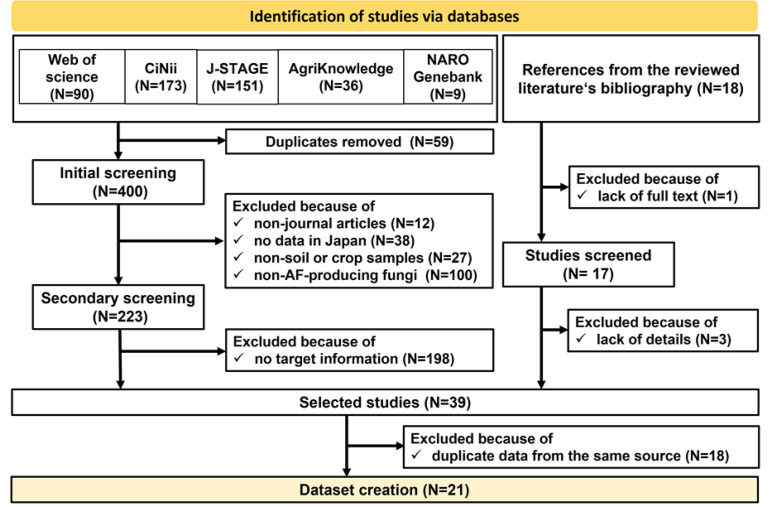
A PRISMA diagram depicting data collection and selection processes. N is the number of studies.

A systematic literature search was conducted using the following five databases (Fig.4). Five databases containing peer-reviewed scientific publications and bulletins of national and prefectural agricultural research stations in Japan.

1) Web of Science (https://www.webofscience.com/wos/woscc/basic-search)
2) CiNii Research(https://cir.nii.ac.jp/)
3) J-STAGE (https://www.jstage.jst.go.jp/browse/-char/ja/)
4) AgriKnowledge, a database for agricultural science and technology of Japan (https://agriknowledge.affrc.go.jp/)
5) NARO Genebank Database, a database containing passport data for a collection of various genetic resources related to agriculture, including plants, microorganisms, and animals (https://www.gene.affrc.go.jp/about_en.php). We used the Microorganism Search System, which allows for searching species, location, properties, and types of collected materials.

We searched five databases using the search terms shown in Table 1. The search terms are a combination of the fungal genus *(Aspergillus),* the name of the mycotoxin (aflatoxin), and the words Japan, distribution, and contamination.

**Table 1:** List of search terms used for each database search, and the number of articles obtained.

Database	Search terms	Results
Web of Science	(aspergillus OR aflatoxins) AND distribution AND Japan NOT gene	90
CiNii Research	(“Asuperugirusu” OR “Afuratokishin”) and(“Osen” OR “Bunpu”) and “Nihon”	173
J-STAGE	Title:“Asuperugirusu” OR Title:“Afuratokishin” AND content:“Bunpu” OR content:“Osen”	151
AgriKnowledge	“Afuratokishin” “Osen” “Nihon”	36
NARO Genebank	Scientific name : *Aspergillus flavus* Scientific name : *Aspergillus parasiticus*	9

*Aspergillus* is referred to as *“Asuperujirusu”* [8,9] or *“Asuperugirusu”* in Japanese [10]. We, therefore, used both *“Asuperujirusu”* and *“Asuperugirusu”* as search terms when searching Japanese databases such as CiNii Research and J-STAGE. Additionally, the terms *“Afuratokishin”, “Bunpu”, “Osen”* and *“Nihon”* were also used as search terms, which mean *“Aflatoxin”, “distribution”, “contamination”*, and *“Japan”*, respectively.

In June 2023, we searched Web of Science and AgriKnowledge using the search terms listed in Table 1, yielding 90 and 36 results, respectively (Fig.4). Additionally, in July 2023, we searched for microorganism genetic resources conserved in the NARO Genebank. As a result, 137 accessions of *Aspergillus flavus* and 25 accessions *of Aspergillus parasiticus* were found. We selected data on fungal strains collected in Japan that had been published in scientific papers, resulting in 9 relevant articles.

In January 2024, we searched CiNii Research and J-STAGE using the search terms in Table 1, yielding 173 and 151 articles, respectively (Fig. 4).

The selected papers from the five databases were gathered and checked for duplicates, resulting in the removal of 59 duplicates and leaving 400 unique papers. They were initially screened based on their type, study site, source materials, and relevance to AF, yielding 223 results. We then further scrutinized the remaining papers for data availability, resulting in 25 remaining papers.

We further reviewed the bibliographies of the 25 remaining articles selected from the five databases and identified 18 additional studies. In the reference list file, these were marked with “⁎” followed by the reference number, with the source reference number indicated in the righthand column of the file. Of these 18 papers, 14 studies met the inclusion criteria, as shown in the right-hand flow of the PRISMA diagram (Fig. 4).

A total of 39 papers (25 papers directly from the database searches and 14 papers from their bibliography) were found relevant to this systematic literature search. We developed a Microsoft Excel spreadsheet and assigned a reference number which is shown in the “Reference_No.” column of the main dataset. In case where multiple samples were available in a single paper, we added multiple rows and assigned a different dataset number to each sample. We examined the content of these papers to identify the original sources of the samples. We found that multiple papers often by the same authors used the same samples, based on matching sampling sites, times, and identified species. These duplicates were indicated in the “Dataset_note” column or labeled with the same dataset number in the main dataset. This scrutiny resulted in the removal of 18 studies.

After removing those duplicated samples, we have developed a dataset containing 137 sets from 21 studies.

### 4.3 Data extraction

#### 4.3.1 Site information

The geographical site information was obtained from the text as much as possible. Where the site information was provided only on the map, we identified the sampling sites using Google Maps. The dataset file also includes latitude and longitude coordinates representing the center of the municipality to facilitate map visualization.

#### 4.3.2 Presence or absence of aflatoxin-producing fungi

We extracted information on the presence or absence of aflatoxin-producing fungi, along with sample sources (e.g., from soil or crop) and taxonomic identification of the fungi. Because presence or absence was reported for individual samples, replication data could not be extracted.

#### 4.3.3 Climatological data

Climate data such as annual mean air temperatures and precipitation were obtained, in principle, from the nearest weather stations operated by the Japan Meteorological Agency (JMA), including AMeDAS (Automated Meteorological Data Acquisition System) stations and JMA weather stations at sites where the site information was available. AMeDAS stations are located approximately 10 to 20 km apart across Japan, with daily data recorded at each station since the late 1970s. JMA weather stations are more sparsely distributed, typically around three stations per prefecture on average, but provide longer-term records. If the nearest station was not selected (e.g. due to differences in geography or altitude), the rationale for selecting an alternative station is described in the meteorological note in the dataset file. In principle, air temperature and precipitation were aggregated over the 10 years preceding the sampling time. If data from that period were unavailable, the average for the oldest available 10-year period was used instead. The dataset includes links to comma separated value (CSV) files containing all weather records at the JMA weather stations or AMeDAS.

## Limitations

1. Detailed information of sampling site was not available as it was not listed.
2. Because specific site information was not available for some samples, climate data could not be linked for those samples. Additionally, as climate data were obtained from the nearest AMeDAS station, the temperature and precipitation data contain some uncertainty due to the difference in conditions between the sampling site and AMeDAS site.

## Ethics Statement

The work does not involve any human subjects, animal experiments, or data collected from social media platforms.

## CRediT Author Statement

**Yuko Tsukada**: investigation, writing- reviewing and editing; Masayo Kushiro: Conceptualization, reviewing & editing. **Hitomi Wakatsuki:** Methodology development, reviewing & editing. **Toshihiro Hasegawa:** Conceptualization, reviewing & editing. **Motoki Nishimori:** Project supervision, reviewing and editing.

## Data Availability

Mendeley DataA dataset of the distribution of aflatoxin-producing fungi in Japan (Original data)

## Footnotes

⁎ Words in parentheses “ ” are Japanese.
